# Principal Component Analysis of Categorized Polytomous Variable-Based Classification of Diabetes and Other Chronic Diseases

**DOI:** 10.3390/ijerph16193593

**Published:** 2019-09-25

**Authors:** Musa Uba Muhammad, Ren Jiadong, Noman Sohail Muhammad, Munawar Hussain, Irshad Muhammad

**Affiliations:** Department of Information sciences and Technology, Yanshan University, Qinhuangdao, Hebei 066000, China; jdren@ysu.edu.cn (R.J.); mn.sohail@stumail.ysu.edu.cn (N.S.M.); munawarjut@yahoo.com (M.H.); ibrahim@stumail.ysu.edu.cn (I.M.)

**Keywords:** diabetes mellitus, cardiovascular problem, data mining, classification, eigenvalues, correlation coefficient, hypertension, PCA, variance

## Abstract

A chronic disease diabetes mellitus is assuming pestilence proportion worldwide. Therefore prevalence is important in all aspects. Researchers have introduced various methods, but still, the improvement is a need for classification techniques. This paper considers data mining approach and principal component analysis (PCA) techniques, on a single platform to approaches on the polytomous variable-based classification of diabetes mellitus and some selected chronic diseases. The PCA result shows eigenvalues, and the total variance is explained for the principal components (PCs) solution. Total of twelve attributes was analyzed with the intention to precise the pattern of the correlation with minimum factors as possible. Usually, factors with large eigenvalues retained. The first five components have their eigenvalues large enough to be retained. Their variances are 18.9%, 14.0%, 13.6%, 10.3%, and 8.6%, respectively. That explains ~65.3% of the total variance. We further applied K-means clustering with the aid of the first two PCs. As well, correlation results between diabetes mellitus and selected diseases; it has revealed that diabetes patients are more likely to have kidney and hypertension. Therefore, the study validates the proposed polytomous method for classification techniques. Such a study is important in better assessment on low socio-economic status zone regions around the globe.

## 1. Introduction

Diagnosis of chronic diseases is essential in the healthcare field as these diseases persist for a long time. The major chronic diseases include diabetes, cardiovascular disease, kidney problem, cancer, and stoke. Classification of the disease helps in taking precautionary actions, and effective treatment at an initial stage found to be helpful for patients [1].

Principal component analysis (PCA) a trendy method for data reduction, found to be a useful step in classification [2]. PCA and machine learning methods were successfully applied in medical domains, for example, in the diagnosis of diabetes aspects, therapy, prognostics of recurrence of breast cancer, localization of a primary tumor, and diagnosis of thyroid diseases. Polytomization of variables may occur in situation categorical variables are of multiple outcomes, for more subjective assessment and evaluation, as the factors scores obtained from binary variables are linearly related [1,2,3]. 

As a chronic disease, diabetes mellitus is assuming pestilence proportion worldwide, by affecting developed and underdeveloped countries at the same time [4]. The most recent prevalence figure published by the International Diabetes Federation is 425 million people living with diabetes mellitus worldwide, with almost 50% non-diagnosed cases [5].

The disease is characterized by high blood glucose. Failure of the pancreas to produce enough insulin and the body’s inefficient use insulin are both pathologic causes of diabetes. Diabetes cannot currently be cured, but only controlled through medication and treatment. Other chronic diseases include hypertension, cardiovascular diseases, kidney problems, and eye problems [6].

Some factors might cause diabetes or increase the risk of the disease. Treatment options can be scientifically advanced on a microscopic and genetic level, through interactions of glucose and insulin in the metabolic system and to macroscopic factors such as social and lifestyle elements, all of which contribute to the disease itself [7]. 

According to the authors of [8,9], obesity is one of the contributory factors for diabetes. Obesity is a situation of having a body mass index (BMI) more than 30. 

Nevertheless, those with a lower BMI but who have a large percentage of body fat, mainly centered on their waist, are also at high risk. Besides this, additional contributory factors of diabetes include high blood pressure history patents, heart attack, or stroke [10,11]. Also, if a family member has had diabetes, the risk is increased [12].

Higher risk of cardiovascular diseases including heart disease, angina and stroke, among others, could be associated with diabetes. As a result of excess glucose in the blood, leaving the body via kidneys may increase risk of kidney problem and related diseases. Kidney problems account for almost 16% of all deaths in diabetics [13].

Eye problem-related diseases, as well as being affected by diabetes and retinopathy, are persistent in patients with long term history of diabetes as the leading cause of preventable loss of sight [14]. 

PCA is a popular technique for data dimension reduction, which, in turn, is a necessary step in classification [15]. By predicting the data onto the dominant eigenvectors, the dimension of the original dataset can be reduced with little or no loss of information.

Asymptotic theory for principal component analysis was developed by [16]. The method demonstrates the uses of principal component analysis. The steps include selecting and measuring a set of variables, preparing the correlation matrix, extracting a set of factors from the correlation matrix, determining the number of factors, rotating the elements, and interpreting the results.

In his book [17], PCA was presented in different fields such as agriculture, biology, ecology, finance, health, taxonomy, and architecture. In the same way, PCA also was applied to multispectral-multimodal optical image analysis for malaria diagnostics [18].

In this paper, we consider an approach that combines data mining techniques and PCA, to approaches polytomous variable-based classification of diabetes mellitus and other chronic diseases. The remaining paper is structured as follows. Section 2 presents the material and methodology; after, Section 3 reviews the results, Section 4 discusses the results, and Section 5 concludes the findings.

## 2. Materials and Methods

### 2.1. Ethical Approval

The Research approved by the Natural Science Foundation of China Hebei province, the Yanshan University ethics committee. Also, all procedures performed in studies involving human participants were under the 1964 Helsinki declaration.

Besides, ethical clearance was also granted by the seven northwestern states of Nigeria. From their respective Ministry of Health; Jigawa, Kano, Kaduna, Katsina, Kebbi, Sokoto, and Zamfara States, with grant codes FMC/BKD/CLN/HREC/138, MOH/ADM/744/VOL.I/558, MOH/OH/797/T.I/456, MOH/ADM/SUB/1152/214, MOH/SUB/4679/I, SMH/1580/V.IV, and HSMB/SUB/540/VOL.I, respectively. All participants provided written informed consent after having all procedures explained to them.

### 2.2. Data Source

Real-life data were collected from both primary and secondary sources, in the northwestern states of Nigeria. The author distributes questionnaires to both diabetes and other chronic diseases patients. He also asked the verbal interview to those who could not write, with the help from the hospital staff. 

Some part of the hospital’s record related to diseases symptoms and their complications also used. The dataset comprises 281 observations and 34 attributes for this particular research.

### 2.3. Data Description

The dataset used for this research purpose contains 281 observations and 34 attributes on the diabetes mellitus and other chronic diseases patients. 

The attributes’ information is as follows.

*AGE*—Patient’s age (numeric).

*GLU*—Patient’s glucose level (numeric with range of 3.9 to 7.2 mmol/L normal and >7.2 mmol/L diabetic).

*DBP*—Patient’s diastolic blood pressure (numeric: <80 mmHg normal, 80 to 120 mmHg hypertensive and >120 mmHg crisis).

*BMI*—Patient’s body mass index (numeric: <18.5 kg/msq underweight, from 18.5 to 25 kg/msq normal, 25 to 30 kg/msq overweight, and >30 kg/msq obese).

*WGT*—Patient’s weight (numeric).

*OCP*—Patient’s occupation status (numeric: 1—student, 2—housewife, 3—others, 4—civil servant).

*SEX*—Patient’s sex (categorical: M—male and F—female).

*DIT*—Diet took by the patient (categorical: NBD—not a balanced diet; BLD—balanced diet).

*MST*—Patient’s marital status (categorical: SG—single, MR—married, SP—separated, DV—divorced, and WD—widowed).

*DCD*—Patient’s diabetes condition (categorical: IND—insulin dependent, NID—non-insulin-dependent and GTD—gestational).

*RSB*—Patient’s residential Suburb (categorical: VL—village, TW—town and CY—city).

*LOE*—Patient’s level of education (categorical: NE—not go to school, PE—primary school, HE—high school, CE—college/university, PG—postgraduate, and IE—Islamic school). 

*EXT*—Excessive thirst (binary yes or no).

*FRU*—Frequent urination (binary yes or no).

*WLG*—Weight loss or gain (binary yes or no).

*FLS*—Flulike symptoms (binary yes or no).

*BRV*—Blurred vision (binary yes or no).

*IRT*—Irritability (binary yes or no).

*SHC*—Slow healings on cut or bruise (binary yes or no).

*TLF*—Tingling or loss of feeling in hand or feet (binary yes or no).

*RIV*—Recurring infection on gum or skin (binary yes or no).

*RIV*—Recurring vaginal/bladder infection (binary yes or no).

*SFH*—Swelling on the ankle, fit or hand (binary yes or no).

*VMT*—Vomiting (binary yes or no).

*FTG*—Fatigue (binary yes or no).

*SCE*—Spiders, cobwebs or specks in the eye (binary yes or no).

*DRV*—Dark streaks or red that blocks vision (binary yes or no).

*EYP*—Eye pain (binary yes or no).

*PCJ*—Pain in chest, jaw, or arm (binary yes or no).

*SOB*—Shortness of breath (binary yes or no).

*SWG*—Swelling (edema) (binary yes or no).

*NRV*—Nervousness (binary yes or no).

*HIT*—Heat intolerance (binary yes or no).

*SBF*—Slowing in body function (binary yes or no).

### 2.4. Diabetes Mellitus

Diabetes is a chronic disease that occurs either when the pancreas does not produce enough insulin or when the body cannot efficiently use the insulin it produces. Insulin is a hormone that controls blood sugar. Hyperglycemia or higher blood sugar is a common effect of uncontrolled diabetes and, over time, leads to severe damages in the entire human body’s systems [19]. 

The diabetes conditions include type I diabetes, also known as “Insulin-dependent” (IND). It is characterized by beta cell destruction caused by an autoimmune process, typically leading to absolute insulin deficiency. Ultimately, all IND patients will require insulin remedy to maintain normglycemia.

Type II diabetes is also known as “Non-insulin-dependent” (NID). The most common form of diabetes mellitus and is highly associated with a family history of diabetes, older age, obesity and lack of exercise. NID comprises 80% to 90% of all cases of diabetes mellitus. Primarily individuals with Type 2 diabetes exhibit intra-abdominal (visceral) obesity, which is directly related to the presence of insulin resistance, in addition to hypertension and dyslipidemia.

The disorder identified in women who develop IND during pregnancy, and women with undiagnosed asymptomatic NID discovered during pregnancy classified as Gestational Diabetes (GTD) [19,20].

Diagnostic criteria for diabetes: fasting plasma glucose ≥ 7.0 mmol/L or 2-hour post-load plasma glucose ≥ 11.1 mmol/L or Hba1c ≥ 48 mmol/mol. For gestational diabetes: fasting plasma glucose 5.1–6.9 mmol/L or 1-hour post-load plasma glucose ≥ 10.0 mmol/L or 2-hour post-load plasma glucose 8.5–11.0 mmol/L [19]. 

In some countries of low and middle incomes, use the most straightforward test that does not require fasting before taking the test, if 200 or more than 200 mg/dl of blood glucose it probably indicates diabetes but has to be reconfirmed [20]. 

### 2.5. Sampling Procedure

The sampling procedure used in distributing the questionnaires and determining the sample sizes is a probability cluster sampling process at the beginning. The entire populations were divided into clusters, which is allied with a selection of a subset of individuals from the populace to estimate the characteristics of the entire population [21]. All the attributes determine one or more properties of the observable subjects distinguished as independent individuals.

The nonprobability sampling procedure was also applied. It is a process by which a researcher selects a sample based on subjective judgment rather than random selection. Thus, not all the members of the population have the same equal chance of participating in the study, unlike the probabilistic method. 

The northwestern part of Nigeria comprises of seven states. We divide each state into three clusters according to their senatorial zones (i.e., central, north, and south). Own government hospitals were chosen in each group from the six states; whereas, in Kano State, we double the number due to its population. 

This particular research is ongoing from 2017 until 2019. Presently, we collected the data from Jigawa, Kano, Katsina, Zamfara, and some part of Sokoto states. The hospitals covered Ajingi General Hospital (Kano State), Murtala Muhammad Specialist Hospital (Kano State), Abdullahi Wase Specialist Hospital (Kano State), Sir Muhammad Sunusi General Hospital (Kano State), Gaya General Hospital (Kano State), Gwarzo General Hospital (Kano State), Federal Medical Centre Birnin-Kudu (Jigawa State), Hadejia General Hospital (Jigawa State), Gumel General Hospital (Jigawa State), Katsina General Hospital (Katsina State), Daura General Hospital (Katsina State), Malumfashi General Hospital (Katsina State), Gusau General Hospital (Zamfara State), TalataMafara General Hospital (Zamfara State), KauraNamoda General Hospital (Zamfara State), and Bodinga General Hospital (Sokoto State).

### 2.6. Categorization of Continuous Variables

Continuous variables (CVs) encountered in many situations. CVs such as age, body mass index, blood glucose level, blood pressure, and many other things were measured. To relate an outcome variable to a single continuous variable, an appropriate regression model is essential.

CVs may be converted into categorical variables by grouping values into two or more categories. Dichotomize continuous variables occur in one of two possible states, which can label as zero and one, e.g., “improved/not improved”, “completed task/failed to complete the task”, etc. In a situation whereby the grouping of variables is more than two categories, referred to as polytomous continuous variables [22].

There are several advantages of dichotomizing continuous variables, but these no statistical grounds [23]. The most common argument seems to be simplicity. Placing all individuals into two groups is extensively perceived to greatly simplify statistical analysis and lead to straightforward interpretation and presentation of results. 

From the literature point of view, it has observed that dichotomization of variable had been criticized extensively [22,23,24,25,26]. According to the authors of [27], when the real risk increases (or decreases) monotonically with the level of the variable of interest, the apparent spread of risk will increase with the number of groups used. With only two groups, one may seriously underestimate the extent of variation in risk. This means that when individuals are divided into just two groups, considerable variability may subsume within each group. 

**Figure 1:**
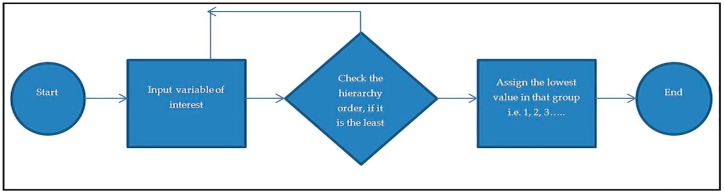
Variable categorization platform.

Therefore, the study adopts the grouping of categorical variables to be polytomous, except where they are naturally dichotomous. The procedures involved presented by the flowchart as in Figure 1.

A proposed polytomous categorization of variables procedures executed in java codes, the process involved in classifying the variables implemented in “if-else-if statement” algorithm. The algorithms perform the tasks and screen each categorical variable before assign to the appropriate group. 

**Figure 2:**
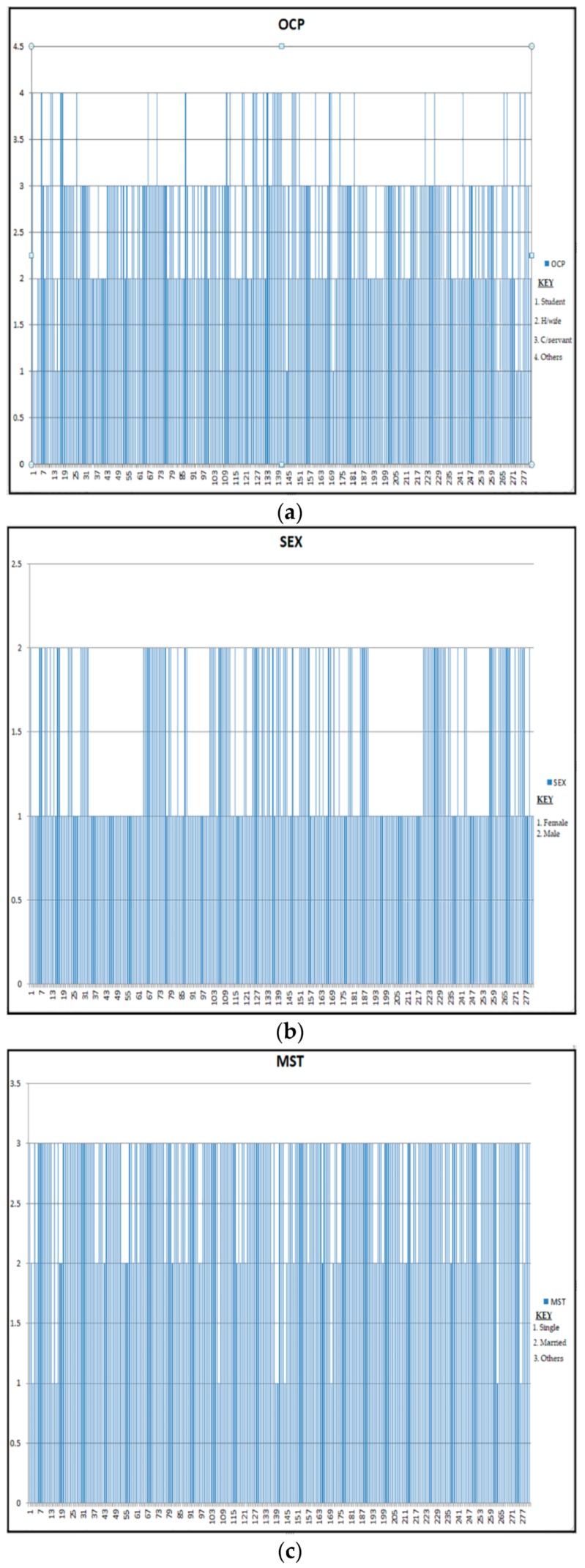

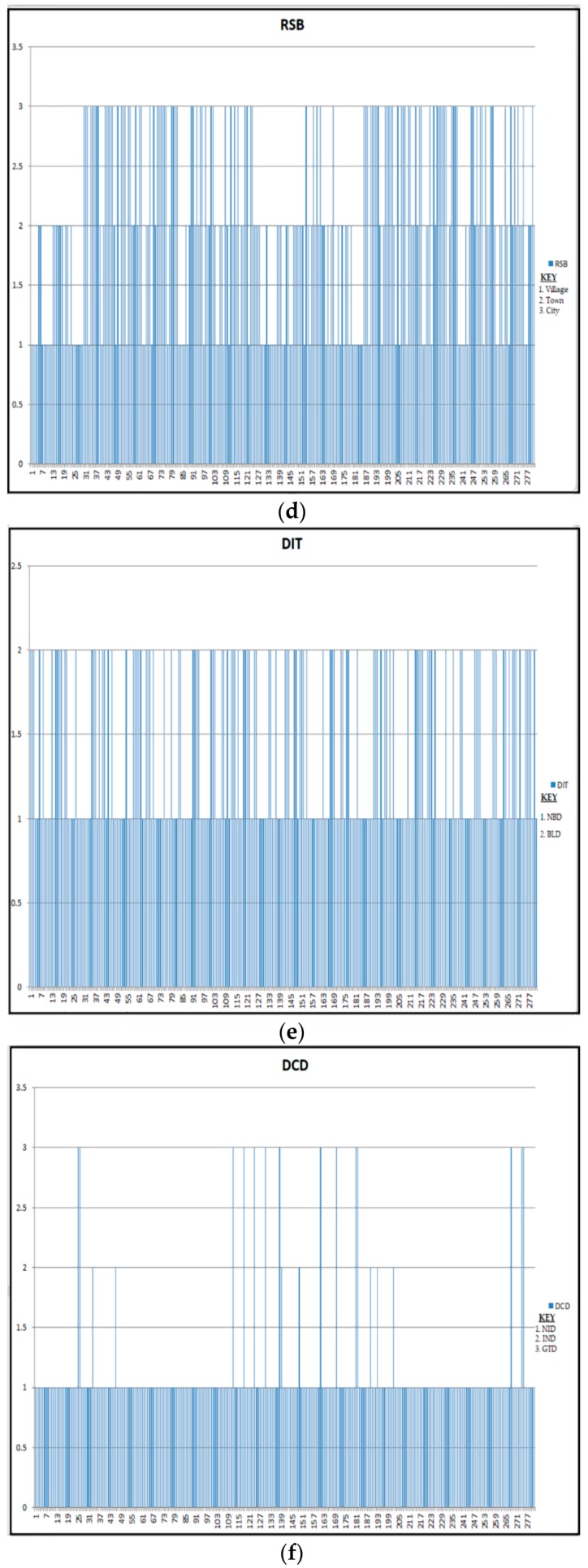

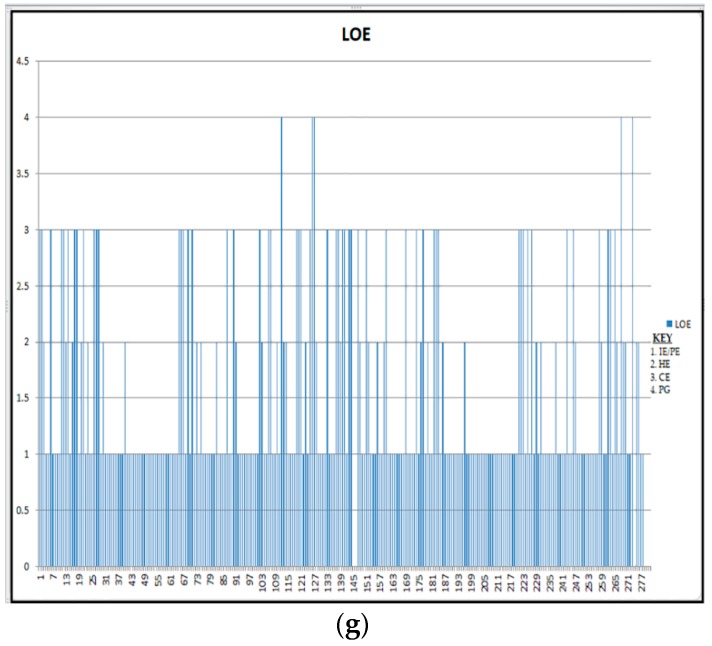
(**a**–**g**) Data flow for the categorized variables of interest.

The study achieved seven if-else-if statements for the classification process by successfully categorizing seven different categorical variables. Data flow for the categorized variables of interest presented below in Figure 2.

### 2.7. Principal Component Analysis

Principal component analysis is a high utility multivariable analysis (MVA) technique used in summarizing the information contained in a continuous multivariable dataset, by reducing the dimension of the dataset without losing useful information. PCA is aimed to identify a hidden pattern in any given dataset, reduce redundancy and eliminate noise. 

PCA works in a highly correlated environment. So, it selects a set of variables that are highly dependent with each other, but at the same time, they are entirely uncorrelated with different subsets of variables which are combined to form a factor. 

The basic idea here is that these newly formed factors drive the fundamental process due to which the variables in the data set are thought to correlate with each other. The specific goals are to summarize patterns of correlations among observed variables, to reduce a large number of observed variables to a smaller number of factors, and to provide an outfitted definition for an underlying process by using observed variables [28]. The PCA model is based on the following model,

(1) $$Y_{ij}=\beta_{i1}X_{1j}+\beta_{i2}X_{2j}+\beta_{i3}X_{3j}+\ldots \ldots \ldots +\beta_{ip}X_{pj}$$

where *i*, *j* = 1, 2, 3, …, *p*.

The correlation matrix for the complete data based on the sizes of some correlation place constraints on the sizes of others. In particular,

(2) $$r_{13}r_{23}=\sqrt{\left(1-r_{13}{}^{2}\right)}\left(1-r_{23}{}^{2}\right)\leq r_{12}\leq r_{13}r_{23}+\sqrt{\left(1-r_{13}{}^{2}\right)}\left(1-r_{23}{}^{2}\right)$$

### 2.8. Software Programming Language 

The programming software used for the experiments are free open programming software for programming, statistics, and graphics. This was developed by Ross Ihaka and Robert Gentleman at the University of Auckland in New Zealand, the name (R) came from their respective first names [29], and can be accessed online via http://www.r-project.org. Also, an object-oriented programming language (java platform) was used in the process of categorizing the variables. 

## 3. Result

In this study, a combine data mining and PCA techniques prompted on the real-life dataset. The dataset of 281 patients suffering from diabetes mellitus and related diseases were collected from the northwestern part of Nigeria. The results presented in both figures and tables below.

Table 1 presents descriptive statistics of some attributes and their prevalence among 281 instances. These are diabetic conditions (DCD) with three different states, average patients’ weight in respect to DCD, and patients’ age grouped with DCD.

Table 2 presents the eigenvalues and the percentages of the explained variances for the principal component analysis results accounted in each principal component (PC). The amount of variation defined by each eigenvalue has shown in the second column. The value 2.264 divided by 12 equals 0.189, or the first eigenvalue explains ~18.9% of the variation. 

The cumulative percentage explained is given in the last column. The first five (5) components out of twelve (12) have their eigenvalue above one (1) and are large enough to be retained. Their explained variances are 18.9%, 14.0%, 13.6%, 10.3%, and 8.6%, respectively, and thus described 65.38% of the total variance.

**Figure 3:**
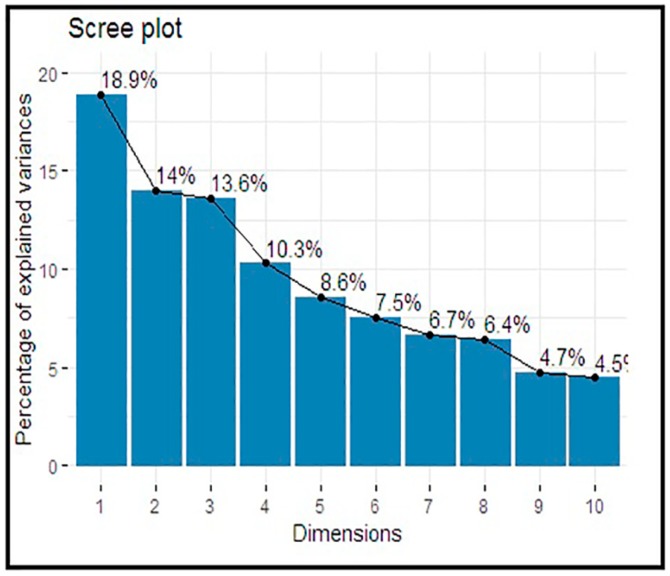
Scree plot for the percentage of explained variances by each component.

Figure 3 represents a Scree plot for the percentage of explained variances by each component. 

**Figure 4:**
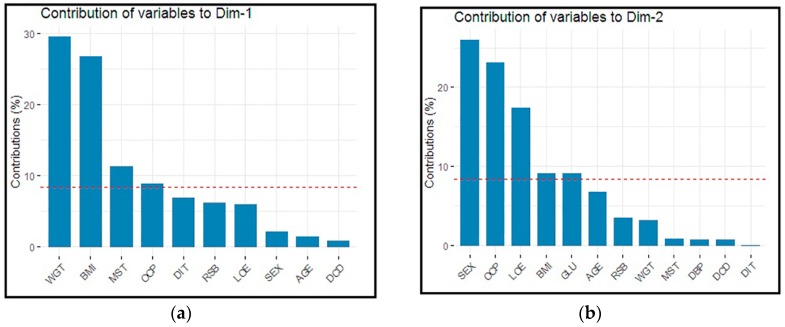
(**a**,**b**) Bar plots for the individual variables contribution to PCs in Dim-1 and Dim-2.

Figure 4a represents bar plots for the individual variables contribution to PCs in dimension one (Dim-1). For example, the variable “WGT” contributed the highest percentage, followed by BMI, etc. Figure 4b represents bar plots for the individual variables contribution to PCs in dimension 2 (Dim-2). For example, the variable SEX contributed the most significant percentage followed by OCP, etc.

**Figure 5:**
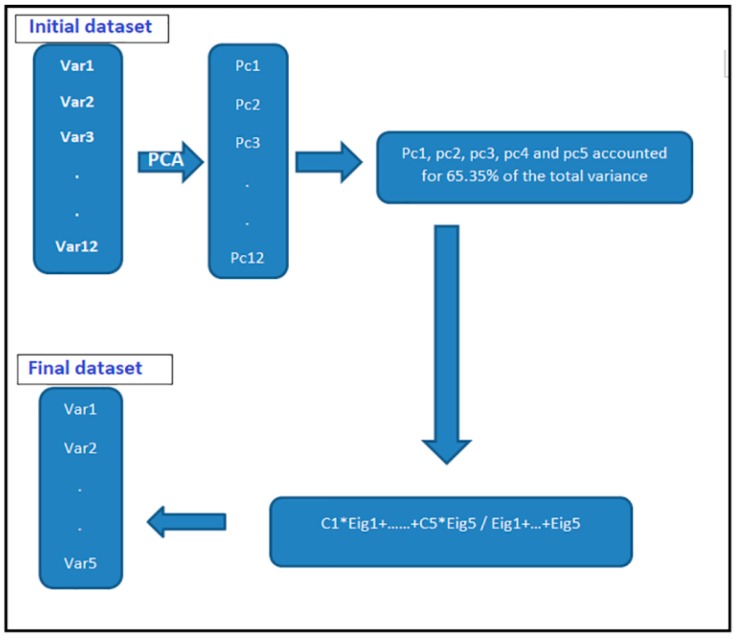
The data reduction process flow.

Figure 5 below, represents a data reduction process flow, at the initial stage, twelve variables entered, and in the final stage, the dataset reduced to five variables.

**Figure 6:**
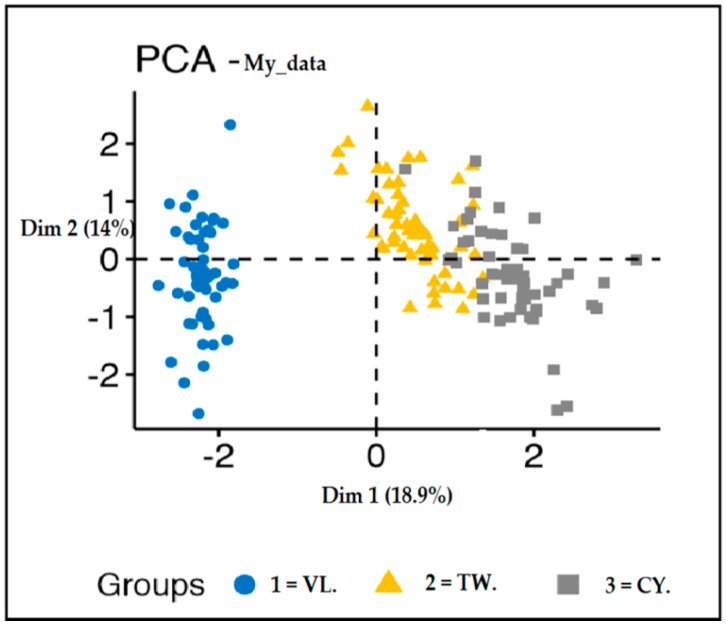
Represents the K-means clusters assessment for the attribute residential suburb. (Legends: VL: Village; TW: Town; CY: City).

Figure 6, below, presents K-means clusters example for the attribute “Residential suburb” classified as Village, Town, and City for the 281 instances. Also, the same applies to the remaining attributes.

Table 3 presents a correlation matrix for the variables involves in the study to check the degree of relationship between them. It was observed that the principal diagonal leading elements are all equals 1. 

Besides, variable BMI and WGT have the highest correlation value of 0.81, indicating strong positive relation. Variable AGE and GLU, SEX and OCP, MST and OCP, LOA and OCP, and SEX and LOE with their respective correlation values of 0.52, 0.39, 0.33, 0.32, and 0.30, respectively, were all are positively correlated.

**Figure 7:**
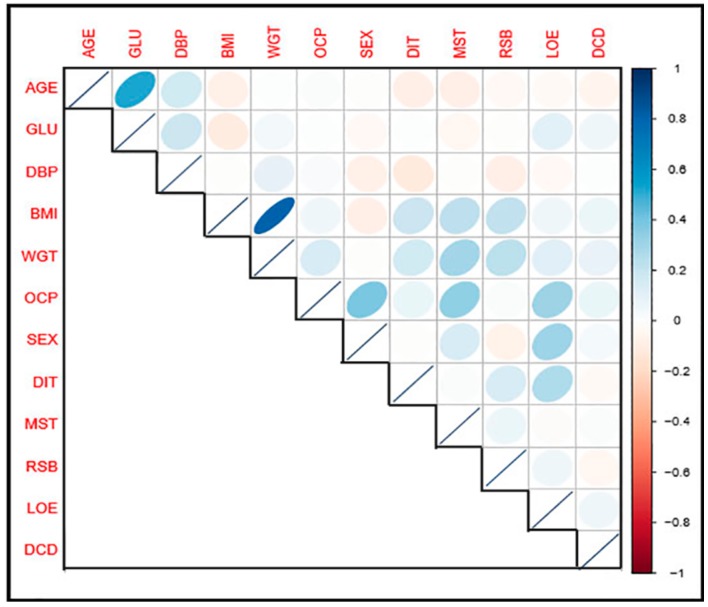
Correlation Matrix.

Figure 7 presents a pictorial representation of a correlation matrix. The darker the color, the more strongly the relationship is, and vice versa.

**Figure 8:**
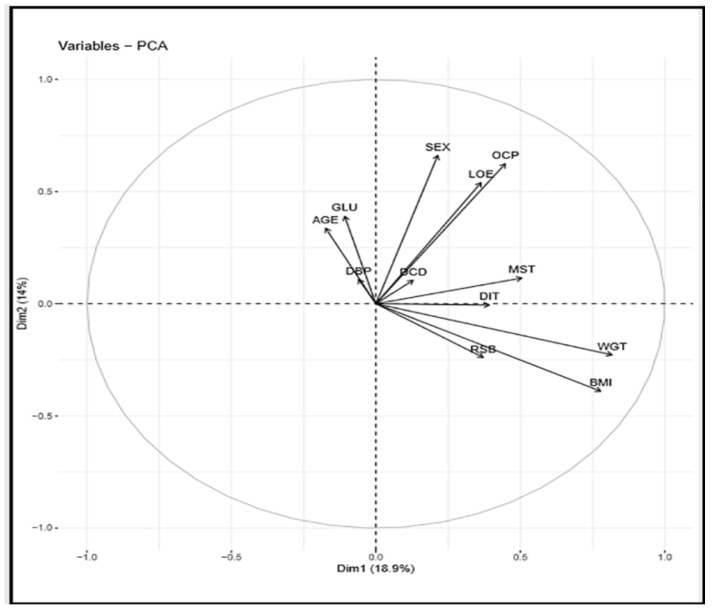
Correlation Circle.

Figure 8 presents a correlation circle between the variables and PCs in dimensions 1 and 2, serving as the coordinate of the variables on the PCs.

Table 4, below, presents a correlation coefficient result for some selected symptoms of diabetes mellitus and other chronic diseases; the first column represents diabetes mellitus symptoms, and the first raw represents symptoms from four different chronic diseases involved in the study. The chronic diseases include high blood pressure, kidney problems, cardiovascular problems, and eye problems. 

It was observed that the symptoms (FTG) related to kidney problems and high blood pressure has a relatively high correlation value of (0.43). Symptom (SOB) related cardiovascular problem, symptom (SCE) related to eye problem and symptoms (NRV) of high blood pressure with their correlation values of (0.42), (0.37), and (0.30), respectively.

On the other hand, the study recorded negative correlations between some symptoms among chronic diseases and diabetes mellitus. Also, symptoms (SFH) related to kidney problems and (DRV) associated with eye problems, with correlation values of (−0.01) and (0.01), respectively, show no relationship.

## 4. Discussion

This study uses java codes in performing the variables categorization by implementing If-else-if statements algorithms to avoid loss of vital information from the dataset. The main strength of this study is the use of a real-life dataset from the authentic hospital records of Nigerian hospitals, which was used to validate all our results.

The outcomes of the categorized variables in Table 2, indicates that this method can successfully be implemented to classify the variables to meaningful clusters. Thus, clusters evaluation has shown in Figure 6, for more subjective assessment and further clinical investigation. Also, such a study is essential in better assessment on low socio-economic status zone regions around the globe. 

On the other hand, from Table 4, it has been observed that there exists a positive relationship between diabetes mellitus symptoms and selected chronic diseases symptoms (FTG) and (SOB) of (0.43). Initially, the experiments were carried out with 281 instances in R and java platforms. The only limitation is the time required to obtain the desired results will be directly proportional to the size of the data use, in case of using a different sample size.

## 5. Conclusions

The study proposed a polytomous method of categorizing variables. The process was applied to classify all the categorical variables of interest in a real-life dataset. The result in Table 2 shows eigenvalues, and the total variance explained for the principal components (PCs) solution. 

There were twelve (12) variables involved in the study, and the aim was to predict the pattern of the correlation with minimum factors as possible. Similarly, the resultant eigenvalues correspond to a different factor, and, usually, factors with large eigenvalues are to be retained. 

It is quite clear that from Table 2 above, the first five (5) components out of twelve (12) have their eigenvalue above one (1) and thus, are large enough to be retained. Their variances are 18.9%, 14.0%, 13.6%, 10.3%, and 8.6%, respectively. These five (5) components describe 65.38% of the total variance.

Besides, from the retrained PCs, the first two PCs were used in the assessment of K-means clusters for the attribute residential suburb.

Moreover, from the correlation results, it has been revealed that diabetes patients are more likely to have kidney or hypertensive problem with the correlation value of (0.43). Rather than having an eye and cardiovascular issues. Therefore, the study validates the proposed categorization method for classification techniques.

## Figures and Tables

**Table 1:** Statistics.

Diabetic Conditions	Instance (*N* = 281)	Weight (Kg)	Age (Year)	Instance (*N* = 281)		
				GTD	IND	NID
GTD	11	65.6 ± 8.96	<20	0	1	0
IND	7	60.5 ± 7.56	20–40	6	1	45
NID	263	62.4 ± 12.85	40–65	5	3	136
			≥65	0	2	82

**Table 2:** Eigenvalues and explained variances for the principal components analysis (PCA) result.

DIM	EV	VP	CVP
Dim 1	2.2637060	18.86	18.86
Dim 2	1.6831058	14.03	32.89
Dim 3	1.6337142	13.61	46.50
Dim 4	1.2350124	10.29	56.79
Dim 5	1.0306272	8.59	65.38
Dim 6	0.9044255	7.54	72.92
Dim 7	0.8012641	6.68	79.60
Dim 8	0.7679526	6.40	86.00
Dim 9	0.5675913	4.73	90.73
Dim 10	0.5353058	4.46	95.19
Dim 11	0.4093681	3.41	98.60
Dim 12	0.1679269	1.40	100.00

Legends: DIM: Dimension; EV: Eigenvalues; VP: Variance percentage; CVP: Cumulative variance percentage.

**Table 3:** For the attributes.

	AGE	GLU	DBP	BMI	WGT	OCP	SEX	DIT	MST	RSB	LOE	DCD
AGE	1.00	0.52	0.17	−0.07	0.01	0.02	−0.01	−0.09	−0.03	−0.03	−0.02	−0.06
												
GLU		1.00	0.18	−0.11	0.04	0.00	−0.03	0.00	−0.05	−0.01	0.11	0.06
DBP			1.00	−0.01	0.09	0.02	−0.08	−0.11	−0.01	−0.09	−0.03	0.01
BMI				1.00	0.81	0.05	−0.09	0.19	0.22	0.22	0.05	0.06
WGT					1.00	0.14	−0.01	0.17	0.29	0.24	0.11	0.08
OCP						1.00	0.39	0.08	0.33	0.02	0.32	0.08
SEX							1.00	−0.01	0.14	−0.07	0.30	0.03
DIT								1.00	0.02	0.14	0.26	−0.02
MST									1.00	0.07	−0.02	0.02
RSB										1.00	0.06	−0.05
LOE											1.00	0.05
DCD												1.00

Legends: AGE: Patients age; GLU: Patients glucose level; DBP: Patients diastolic blood pressure; BMI: Patients body mass index; WGT: Patients weight; OCP: Patients occupation status; SEX: Patients sex; DIT: Diet took by patient; MST: Patients marital status; LOE: Patients level of education; DCD: Patients diabetic condition.

**Table 4:** Results for some selected symptoms of diabetes mellitus about other chronic diseases.

	SFH	VMT	FTG	SCE	DRV	EYP	PCJ	SOB	SWG	NRV	HIT	SBF
**EXT**	0.22	0.21	0.28	−0.06	−0.09	0.03	0.16	0.29	0.23	0.02	−0.09	0.08
**FRU**	0.16	0.07	0.30	0.22	0.08	0.14	0.01	0.27	0.21	0.07	−0.10	−0.05
**WLG**	−0.07	−0.02	0.06	0.23	0.01	−0.03	0.11	0.02	−0.08	0.22	0.11	0.27
**FLS**	0.09	0.20	0.43	0.15	0.14	0.11	0.06	0.42	0.14	0.04	−0.04	0.04
**BRV**	−0.01	0.01	0.03	0.37	0.01	0.09	0.11	0.05	−0.02	0.23	0.20	0.21
**IRT**	0.19	0.09	0.10	0.27	0.23	0.21	0.14	0.06	0.15	0.21	0.18	0.04
**SHC**	0.18	0.16	0.12	−0.02	0.21	0.16	0.08	0.11	0.20	−0.07	−0.08	−0.02
**TLF**	0.31	0.18	0.02	0.08	0.09	0.17	0.25	0.04	0.19	0.30	0.16	0.24
**RIG**	0.13	0.28	0.01	−0.04	0.03	0.03	0.08	0.08	0.07	0.28	0.29	0.34
**RIV**	0.25	0.23	0.04	−0.01	0.02	0.05	0.25	0.06	0.08	0.20	0.27	0.15

Legends: EXT: Patients suffering from excessive thirst; FRU: Patients suffering from frequent urination; WLG: Patients suffering from unexplained weight loss or gain; FLS: Patients suffering from flulike symptoms; BRV: Patients suffering from blurred vision; IRT: Patients suffering from irritability; SHC: Patients suffering from slow healing on cut or bruise; TLF: Patients suffering from tingling or loss of feeling in hand or feet; RIG: Patients suffering from recurring infection on gum or skin; RIV: Patients suffering from recurring vaginal/bladder infection; SFH: Patients suffering from swelling on ankle, feet, or hand; VMT: Patients suffering from vomiting; FTG: Patients suffering from fatigue; SCE: Patients suffering from spiders, cobwebs, or tiny specks in eye; DRV: Patients suffering from dark streaks or a red that blocks vision; EYP: Patients suffering from eye pain; PCJ: Patients suffering from pain in chest, jaw, or arm; SOB: Patients suffering from shortness of breath; SWG: Patients suffering from swelling (edema); NRV: Patients suffering from nervousness; HIT: Patients suffering from heat intolerance; SBF: Patients suffering from slowing in body function.

## References

[1] Uba M.M., Ren J.D., Sohail M.N., Irshad I., Kaife Y. (2019). Data mining process for predicting diabetes mellitus based model about other chronic diseases: A case study of the northwestern part of Nigeria. Healthcare Technol. Lett..

[2] Divya J., Vijendra S. (2018). Feature selection and classification system for chronic diseases prediction review. Egypt. Inform. J..

[3] Fayyad U., Piatetsky-Shapiro G., Smyth P., Uthurusamy R. (1997). Advances in Knowledge Discovery and Data Mining.

[4] Anthonia O.O., Chukwuma E. (2014). Diabetes mellitus in Nigeria: Past, Present and Future. World J. Diabetes.

[5] International Diabetes Federation (2017). Diabetes Atlas.

[6] Sohail M.N., Ren J., Muhammad M.U. (2019). A Euclidean Group Assessment on Semi-Supervised Clustering for Healthcare Clinical Implications Based on Real-Life Data. Int. J. Environ. Res. Public Health.

[7] Sohail N., Jiadong R., Uba M., Irshad M., Khan A. (2018). Classification and cost-benefit Analysis of Diabetes mellitus Dominance. Int. J. Comput. Sci. Netw. Secur..

[8] Chan J., Rimm E., Colditz G., Stampfer M., Willett W. (1994). Obesity, fat distribution, and weight gain as risk factors for clinical diabetes in men. Diabetes Care.

[9] Resnick H., Valsania P., Halter J., Lin X. (2000). Relation of weight gain and weight loss on subsequent diabetes risk in overweight adults. J. Epidemiol. Commun. Health.

[10] Sernyak M., Leslie D., Alarcon R., Losonczy M., Rosenheck R. (2002). Association of diabetes mellitus with use of atypical neuroleptics in the treatment of schizophrenia. Am. J. Psychiatry.

[11] Dixon L., Weiden P., Delahanty J., Goldberg R., Postrado L., Lucksted A., Lehman A. (2000). Prevalence and correlates of diabetes in national schizophrenia samples. Schizophr. Bull..

[12] Sohail M.N., Ren J., Muhammad M.U., Irshad M., Bilal M., Usman A., Rizwan T. (2018). Forecast Regression analysis for Diabetes Growth: An inclusive data mining approach. Int. J. Adv. Res. Comput..

[13] Morrish N., Wang S.L., Stevens L., Fuller J., Keen H. (2001). Mortality and causes of death in the who multinational study of vascular disease in diabetes. Diabetologia.

[14] Bunce C., Wormald R. (2006). Leading causes of certification for blindness and partial sight in England & Wales. BMC Public Health.

[15] Noman S.M., Jiadong R., Uba M.M., Irshad M., Iqbal W., Arshad J., John A.V. (2019). A hybrid Forecast Cost Benefit Classification of diabetes mellitus prevalence based on epidemiological study on Real-life patient’s data. Nat. Sci. Rep..

[16] Anderson T.W. (1963). Asymptotic theory for principal component analysis. Ann. Math. Stat..

[17] Sanguansat P. (2012). Principal Component Analysis: Multidisciplinary Applications.

[18] Omucheni D.L., Kaduki K.A., Bulimo W.D., Angeyo H.K. (2014). Application of principal component analysis to multispectral-multimodal optical image analysis for malaria diagnostics. Malar. J..

[19] American Diabetes Association (2018). Classification and Diagnosis of Diabetes: Standard of medical. Care Diabetes.

[20] Habtamu W.B. (2015). Classification, Pathophysiology, Diagnosis and Management of Diabetes Mellitus. J. Diabetes Metab..

[21] Singh A.S., Masuku M.B. (2014). Sampling techniques and determination of sample size in applied statistics research: An overview. Int. J. Econ. Commer. Manag..

[22] Royston P., Altman D.G., Sauerbrei W. (2006). Dichotomizing continuous predictors in multiple regression: A bad idea. Stat. Med..

[23] MacCallum R.C., Zhang S., Preacher K.J., Rucker D.D. (2002). On the practice of dichotomization of quantitative variables. Psychol. Methods.

[24] Lagakos S.W. (1988). Effects of mismodelling and mismeasuring explanatory variables on Tests of their association with a response variable. Stat. Med..

[25] Faraggi D., Simon R. (1996). A simulation study of cross-validation for selecting an optimal cutpoint in univariable survival analysis. Stat. Med..

[26] Austin P.C., Brunner L.J. (2004). Inflation of the type I error rate when a continuous confounding variable is categorized in logistic regression analyses. Stat. Med..

[27] Breslow N.E., Day N.E. (1987). Statistical Methods in Cancer Research.

[28] Uba M.M., Ren J.D., Sohail M.N., Irshad I., Musavir B., Osi A.A. (2018). A logistic regression modelling on the prevalence of diabetes mellitus in the northwestern part of Nigeria. Univ. Benin J. Stat. Niger..

[29] Codomy R. (2015). The Ultimate Crash Course to Learning the Basics of R in No Time. Learn R in a Day.

